# An abdominal wall neuroendocrine tumor of unknown primary origin: A case report and review of the literature

**DOI:** 10.1002/cnr2.1610

**Published:** 2022-02-10

**Authors:** Olivia Jagiella‐Lodise, Valerie Jagiella, Evan Weitman

**Affiliations:** ^1^ Royal College of Surgeons in Ireland Dublin Ireland; ^2^ Digestive Healthcare of Georgia Atlanta Georgia USA; ^3^ Piedmont Atlanta Hospital, Division of Surgical Oncology & HPB Surgery Atlanta Georgia USA

**Keywords:** carcinoid, metastasis, peritoneum

## Abstract

**Background:**

Neuroendocrine tumors (NETs) are neoplasms that arise from cells of the endocrine and nervous system. NETs, often found in the gastrointestinal tract, can be found anywhere in the body, and have metastatic potential. NETs occasionally present with metastatic disease without an identifiable primary tumor.

**Case:**

A 79‐year‐old female patient presented with an abdominal wall mass. Percutaneous biopsy was consistent with a NET. Preoperative endoscopy and PillCam were unremarkable. PET Dotatate demonstrated uptake in the abdominal wall as well as vague uptake in the pelvis. Intraoperatively, we identified a suspicious nodule on the sigmoid colon, which was consistent with a drop metastasis on final pathology.

**Conclusion:**

In this case report we present a patient with a NET metastasis to the abdominal wall without a known primary site. This case highlights the limitations of endoscopy and imaging in the workup of metastatic NETs. Additionally, this is a novel case report of a metastatic NET to the abdominal wall without an identifiable primary site.

## 1 INTRODUCTION

Neuroendocrine tumors (NETs) are rare neoplasms arising from cells of the endocrine and nervous system.^1^ NETs most commonly develop in the gastrointestinal tract but can be found anywhere in the body and are often identified incidentally on imaging.^1^, ^2^, ^3^, ^4^ The metastatic potential of NETs can vary depending on the primary location of the tumor. Additionally, NETs seen in association with familial disorders (i.e., pheochromocytomas and paragangliomas in MEN syndromes) typically having a greater metastatic potential than sporadic NETs.^5^ Occasionally, NETs will present as metastatic disease without an identifiable primary tumor location. There are case reports of metastatic NETs to the pancreas and breast without an identifiable primary site; however, our case report is unique as there are no such reported cases with abdominal wall metastases.^1^, ^6^

Clinical workup for NETs includes laboratory studies, imaging studies and comprehensive endoscopy. Chromogranin A, mTOR, CDKN1B, and circulating tumor cell levels have all shown prognostic value for patients with NETs.^5^ On CT imaging, NETs typically enhance with intravenous contrast in the arterial phase and washout during the delayed portal venous phase.^7^ Pan‐endoscopy, CT, magnetic resonance imaging (MRI), Octreoscan and PET Dotatate imaging have been utilized for the localization of NETs.^4^, ^8^, ^9^, ^10^, ^11^, ^12^, ^13^ PET Dotatate has now emerged as the gold standard for imaging of patients with NETs,^11^, ^13^ altering diagnosis and management of one‐third of NET patients,^9^ and increasing the sensitivity of computed tomography (CT) by 50%.^14^

Surgery is the mainstay of treatment for resectable NETs and also has a prominent role in metastatic NETs as it can improve survival.^5^ Unfortunately, many patients who undergo resection of metastatic NETs develop clinical recurrence.^3^, ^5^ The prognosis and management of metastatic NETs is impacted by the primary tumor location as well as the histologic grade of the tumor.^5^ A survival benefit has been demonstrated after debulking of at least 70% of well‐differentiated NETs involving liver metastases when compared to patients treated non‐operatively.^15^

Historically, exploratory surgery has been useful to ultimately identify the primary tumor in cases that were unidentifiable on initial workup.^3^, ^16^, ^17^, ^18^, ^19^ Systemic therapy is typically utilized either before or after surgery in the setting of metastatic disease.^2^, ^12^, ^18^, ^20^, ^21^, ^22^ Somatostatin analogues, such as octreotide and lanreotide, are highly utilized in the management of metastatic NETs.^5^

## 2 CASE REPORT

A 79‐year‐old female patient was referred to surgery for evaluation of a suspicious periumbilical mass with vague abdominal discomfort. She had previously been seen by hepatology for liver cysts and was followed with surveillance CT, which incidentally identified a 4 cm abdominal wall mass. She had no other pertinent medical history.

**FIGURE 1:**
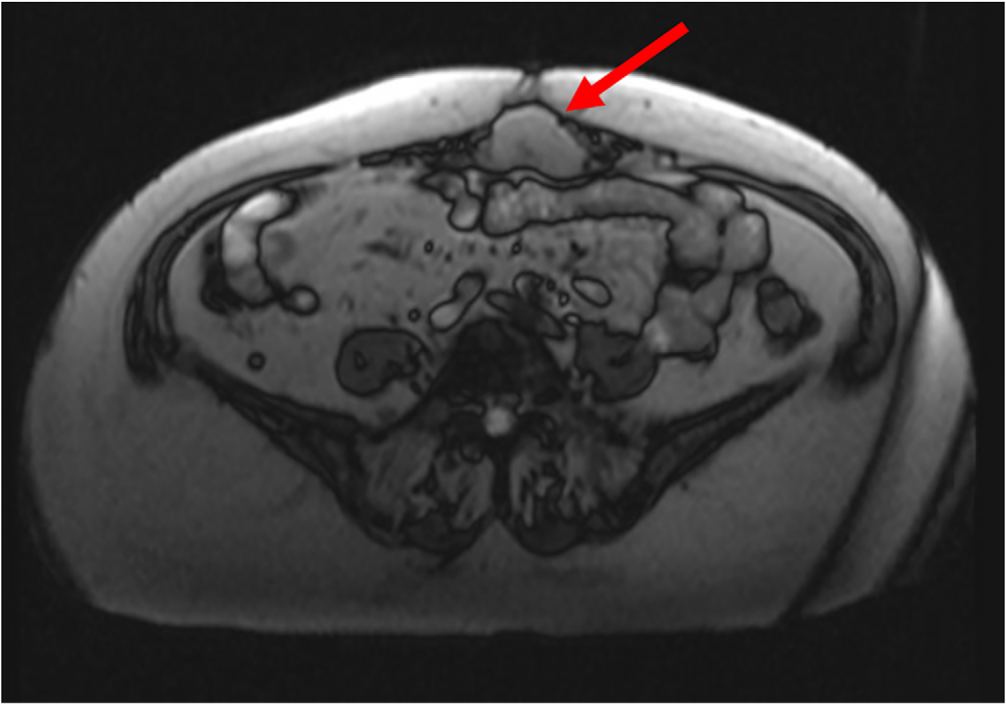
T2‐weighted magnetic resonance imaging (MRI) abdomen images. Red arrow indicates abdominal wall neuroendocrine tumor (NET)

Follow up ultrasound, colonoscopy, and MRI further characterized a 3.8 × 4.1 × 2.5 cm hypoechoic abdominal wall mass (see Figure 1). CT‐guided core needle biopsy was consistent with a well‐differentiated NET. Immunohistochemistry staining was positive for AE1/AE3, synaptophysin, chromogranin and CD56, and negative for beta‐catenin, HMB‐45, desmin, SMA, and inhibin. Ki‐67 proliferation index was estimated to be 3%–5%.

**FIGURE 2:**
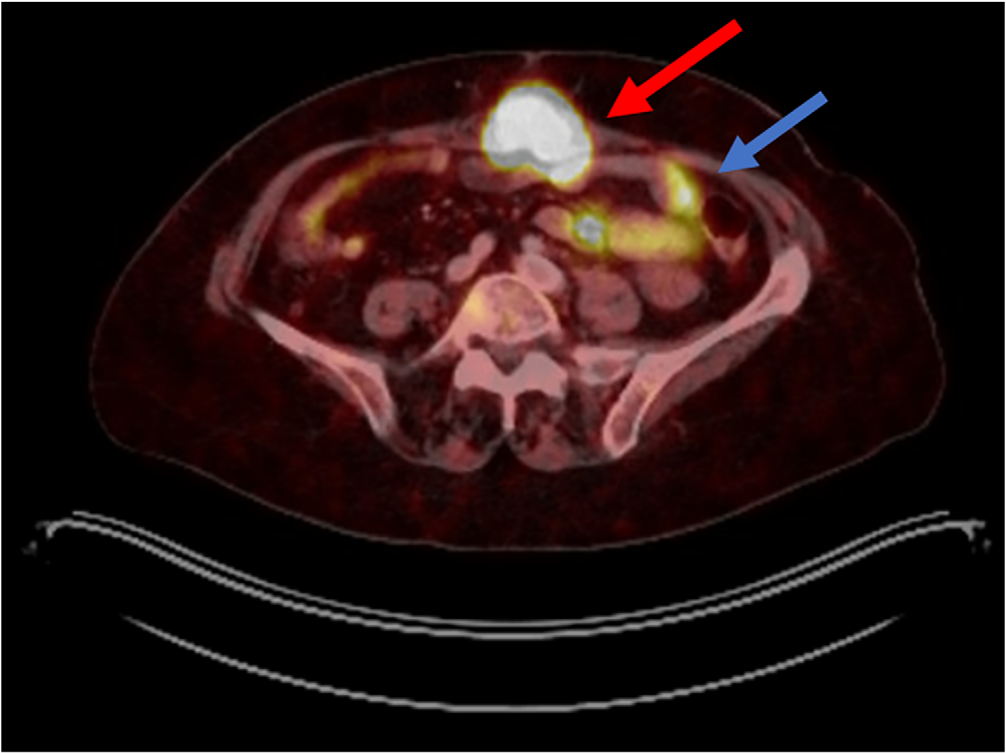
Positron emission tomography (PET) Dotatate images. Red arrow indicates abdominal wall neuroendocrine tumor (NET). Blue arrow indicates vague uptake around sigmoid colon

The patient subsequently underwent EGD, colonoscopy and a PillCam study, all of which were unremarkable. Positron emission tomography (PET) Dotatate imaging demonstrated uptake in the abdominal wall as well as uptake in the pelvis adjacent to the sigmoid colon (see Figure 2).

Given her oligometastatic presentation and lack of an identifiable primary tumor we proceeded with exploratory surgery with planned resection of the abdominal wall tumor. Intraoperatively the abdomen and pelvis were fully explored. The abdominal wall mass was resected. The small bowel was entirely unremarkable on inspection and palpation. A tumor implant was identified on the sigmoid colon and a partial thickness colon resection was performed. The partial thickness colotomy was repaired primarily. A cholecystectomy as well as an appendectomy were additionally performed at this time. Final surgical pathology was consistent with NETs in both the abdominal wall and sigmoid colon specimens. The sigmoid colon specimen demonstrated serosal involvement without mucosal involvement. Surgical margins were all negative. The appendix was unremarkable on final pathology. Her sigmoid lesion was felt to be more consistent with a drop metastasis due to the lack of mucosal involvement on final pathology and her unremarkable preoperative colonoscopy.

Postoperatively, the patient was seen by medical oncology and started on lanreotide. Three and six‐month follow‐up imaging has been unremarkable for any evidence of recurrence.

## 3 DISCUSSION

NETs often originate in the gastrointestinal tract and metastatic foci are commonly identified in the liver as well as the small intestine, pancreas, lung, bone, and brain.^2^, ^3^, ^8^, ^21^, ^23^ Resection of the primary tumor can improve survival in the setting of liver metastases, with or without resection of metastatic disease. One retrospective review demonstrated a median overall survival of 38 versus 10 months for patients with resected versus non‐resected primary NETs in the setting of unresected liver metastases.^24^ Additionally, the primary tumor location has an impact on survival, including in the setting of liver metastases. Specifically, small bowel NETs with metastases demonstrate a better overall survival when compared to gastric and rectal NETs with metastases.^25^ Systemic treatment with either somatostatin analogues as well as chemotherapy is typically utilized for the management of unresectable metastatic NETs.^1^, ^19^, ^26^

In select cases, liver transplantation can be an option for unresectable liver metastases. The Milan selection criteria for patients with liver metastases from NET has been well characterized and identifies a subgroup of patients most likely to demonstrate a long‐term benefit from liver transplantation. The Milan NET criteria include: 1) low grade histology, 2) primary tumor drained by the portal system (i.e., small bowel NETs), 3) tumor burden involving <50% of the liver, and 4) at least 6 months of documented stable disease prior to consideration for transplant.^27^

Treatment guidelines for metastatic NETs of unknown primary tumor location are not well‐established. When histopathology is consistent with well‐differentiated disease, treatment typically involves surgical resection. In such cases, management still often incorporates systemic therapy involving somatostatin analogues, immunosuppression, and/or chemotherapy.^1^, ^8^ When histopathology is consistent with a poorly‐differentiated NET/high‐grade neuroendocrine carcinoma, treatment often involves platinum agents and etoposide.^8^ Cholecystectomy is typically recommended in patients with metastatic NET as treatment with somatostatin analogues can predispose to cholestasis and cholecystitis. Appendectomy is also indicated during surgery for NETs as it is often an occult primary source for NETs.

Multiple case reports have similarly characterized NET metastases to various intramuscular locations; however, in each of those cases a primary NET was identified. The site of metastasis in these cases was most commonly to the lower extremities and the primary tumor location was most frequently identified in the ileocecal region.^28^, ^29^ A few cases have been reported with unusual presumed primary NET locations, including the breast and the femoral sheath.^6^, ^30^

The combination of preoperative imaging with PET Dotatate and exploratory surgery has dramatically improved identification of the primary NET tumor location.^3^, ^4^, ^8^, ^9^, ^11^, ^13^, ^14^, ^17^, ^18^ In one retrospective study on initially occult primary NETS, 64% were identified on further preoperative imaging and 89% were identified at the time of surgery.^3^ However, in many cases the primary tumor location still goes unidentified despite preoperative imaging and exploratory surgery.

A recently developed gene assay has shown early promise in identifying NETs with an unknown primary site. The 92‐gene assay identifies a molecular determinant of NET tumor type/subtype. Specifically, the molecular assay classifies NETs into distinct subtypes, such as pancreas (i.e., pancreatic islet cell carcinoma), skin (i.e., Merkel cell carcinoma), lung (i.e., small cell lung carcinoma), thyroid (i.e., thyroid medullary carcinoma) and adrenal gland (i.e., pheochromocytomas) tumors.^4^

This case report describes a unique presentation of a metastatic NET with an unknown primary and highlights the limitations of PET Dotatate imaging in the workup of metastatic NETs. Long‐term follow up of our patient as well as other patients with unknown primary NETs will hopefully elucidate optimal management approaches for future such NETs patients.

## 4 CONCLUSION

NETs can present myriad clinical challenges due to diagnostic limitations as well as the variability of disease manifestation. Current diagnostic workup of NETs includes PET dotatate imaging, pan‐endoscopy, and surgery; however, this approach does not always localize the primary tumor. Our case report highlights such a clinical conundrum and describes how we proceeded with management. Our patient underwent complete resection of all identifiable gross disease followed by adjuvant treatment with a somatostatin analogue. Her ultimate outcome will likely be driven by her disease biology, as prognosis is highly impacted by histologic grade in NETs. The expression “Biology is King” is often invoked in cancer care. NETs are exemplary in this respect, as well‐differentiated NETs can often behave indolently when compared to poorly differentiated NETs. As diagnostic and therapeutic modalities continue to evolve, hopefully the prognosis of patients with NETs will continue to improve as well.

## CONFLICT OF INTEREST

The authors have stated explicitly that there are no conflicts of interest in connection with this article.

## ETHICS STATEMENT

The Piedmont Healthcare Institutional Review Board has determined this project does not meet the definition of human subject research under the purview of the IRB according to federal regulations.

## AUTHOR CONTRIBUTIONS

**Olivia Jagiella‐Lodise:** Data curation (equal); writing – original draft (equal). **Valerie Jagiella:** Conceptualization (equal). **Evan Weitman:** Conceptualization (equal); formal analysis (equal); supervision (equal); writing – original draft (equal); writing – review and editing (equal).

## Data Availability

This is a retrospective analysis on the de‐identified data of a single patient. As such, this project does not have potential impact on the established body of knowledge in this area. Additionally, a signed privacy authorization is not required as the physician will not include HIPAA identifiers in the submission for publication.
